# Effect of a self-help group intervention using Teaching Recovery Techniques to improve mental health among Syrian refugees in Norway: a randomized controlled trial

**DOI:** 10.1186/s13033-022-00557-4

**Published:** 2022-09-06

**Authors:** Wegdan Hasha, Jannicke Igland, Lars T. Fadnes, Bernadette N. Kumar, Unni M. Heltne, Esperanza Diaz

**Affiliations:** 1 Department of Global Public Health and Primary Care, University of Bergen, Årstadveien 17, 5009 Bergen, Norway; 2 Department of Addiction Medicine, Haukeland University Hospital, Bergen, Norway; 3 Unit for Migration and Health, Norwegian Institute of Public Health, 222 Skøyen, 0213 Oslo, Norway; 4 Centre for Crisis Psychology, Møllendalsbakken 9, 5020 Bergen, Norway

**Keywords:** Refugees, Intervention, Teaching recovery techniques, Randomized controlled trial

## Abstract

**Background:**

Mental health symptoms among refugees are common, often related to chronic pain disorders, and their management is usually challenging. Studies evaluating the effect of group therapies among adult refugees to improve mental health symptoms are scarce.

**Aims:**

To assess the effect of Teaching Recovery Techniques (TRT) on mental health and to reduce pain disorder among adult Syrian refugees.

**Method:**

A randomized controlled trial was designed to study the effect of a self-help group intervention using TRT. The outcomes, mental health symptoms measured by Impact of Event Scale-Revised (IES-R) and General Health Questionnaire (GHQ-12) and chronic pain measured by Brief Pain Inventory (BPI), were reported as regression coefficients (B) with 95% confidence intervals.

**Results:**

Seventy-six adults participated: 38 in the intervention and 38 in the control groups. Intention-to-treat analyses showed a significant effect on general mental health as measured by GHQ-12 with B (95% CI) of -3.8 (-7.2, -0.4). There was no effect of TRT on mental health when assessed by IES-R (-1.3 (-8.7, 6.2)) or on pain levels assessed by BPI (-0.04 (-4.0, 3.9)).

**Conclusions:**

This self-help group intervention significantly improved general mental health symptoms among adult refugees but had no effect on trauma symptoms or chronic pain. Higher participation rates might be necessary to achieve the full potential of TRT.

*Trial registration:* The trial was registered with Clinical Trials.gov at https://clinicaltrials.gov/ct2/show/NCT03951909. To include user participation in the design of the interventions, the study was retrospectively registered on 19 February 2019.

**Supplementary Information:**

The online version contains supplementary material available at 10.1186/s13033-022-00557-4.

## Introduction

The war in Syria has resulted in about 5.6 million international refugees and 6.2 million internally displaced persons, with over a million reaching Europe in 2015 [1, 2], becoming the largest group of refugees in several European countries like Norway [3].

Forced migration because of war and other hazards, as well as acculturation stress in a different society, can have an adverse effect on the health of refugees, leading to mental health problems such as depression, anxiety, or post-traumatic stress disorder (PTSD) [4–9]. Chronic pain is also a common symptom among the refugee population that not only affects everyday life, but also becomes a complicating factor in the treatment of those with co-occurring mental health symptoms [10, 11]. Furthermore, the severity of PTSD symptoms has been found to be associated with increased pain intensity [12, 13]. Our research group has previously reported that the experiences of trauma among Syrian refugees in Lebanon and Norway were associated with both chronic pain and symptoms of depression, anxiety, and PTSD [14] and that poor mental health among participants in the study was associated with chronic pain after one year living in Norway [15].

Many European countries provide universal health coverage to refugees and attempt to provide equitable access to quality health-care services once the refugees are established in the host country. Research on the most suitable treatment options for adult refugees suffering from mental health disorders is limited. Available options of adapted health care services are few as are options for addressing special needs. Therefore, the availability and quality of the health services accessed by this population are greatly compromised [16]. Research from the United Kingdom suggests that secondary care services are often only available to those with a formal diagnosis. Thus far, group interventions conducted for those individuals experiencing mental health symptoms without a formal diagnosis have limited evidence of effectiveness [17, 18]. However, even refugees who do not have enough symptoms for a formal mental health diagnosis may still experience symptoms such as nightmares or flashbacks, often linked to somatic pain, which go unmanaged and may impede acculturation in the host country [6].

There is a need for evidence-based treatments for mental health symptoms among adult refugees [19]. Teaching Recovery Techniques (TRT) is a group intervention previously designed for children 8–18 years old by the Children and War Foundation in Norway to meet the needs of children exposed to war and requiring mental support [20, 21]. TRT is based on the principles of cognitive behavioural therapy (CBT) and evidence-based methods to treat trauma. It has helped reduce mental health symptoms and PTSD among traumatized refugee youths in Sweden and Palestine [22, 23]. The method has not yet been standardized or evaluated for adults, but as one of the very few self-help group interventions targeting refugees with both established diagnoses and symptoms and based on clinical experience, TRT was chosen to be evaluated for adults in this study. Based on our own previous findings of associations of chronic pain and poor mental health, and reports of promising results for psychologically informed physiotherapy [24], we hypothesised that the TRT could also improve chronic pain.

The aim of this study is to assess the effect of TRT on mental health and on pain disorders among adult Syrian refugees.

## Methods

### Study design

This study is part of a 2 × 2 armed randomized control trial (RCT), previously described in a protocol paper [25]. In short, we recruited Syrian adults with either mental health symptoms and/or pain disorders. Participants with predominance of mental health symptoms were allocated to this TRT trial and randomized to either the intervention group or control group, which for ethical reasons received the same intervention (delayed intervention) after six weeks, when the intervention group had completed the intervention. For those presenting both symptoms (pain and mental health), participants who had proportionatly higher levels of psychological symptoms (proportion calculated from a range of 0 to 88 as measured by IES-R) as compared to the pain score levels (proportion calculated from a range of 1 to 40 as measure by BPI) were allocated to the TRT trial. For simplicity, we designate the groups as intervention and control for the rest of the paper. CONSORT guidelines have been used to report this trial [26].

### Participants

**Fig. 1:**
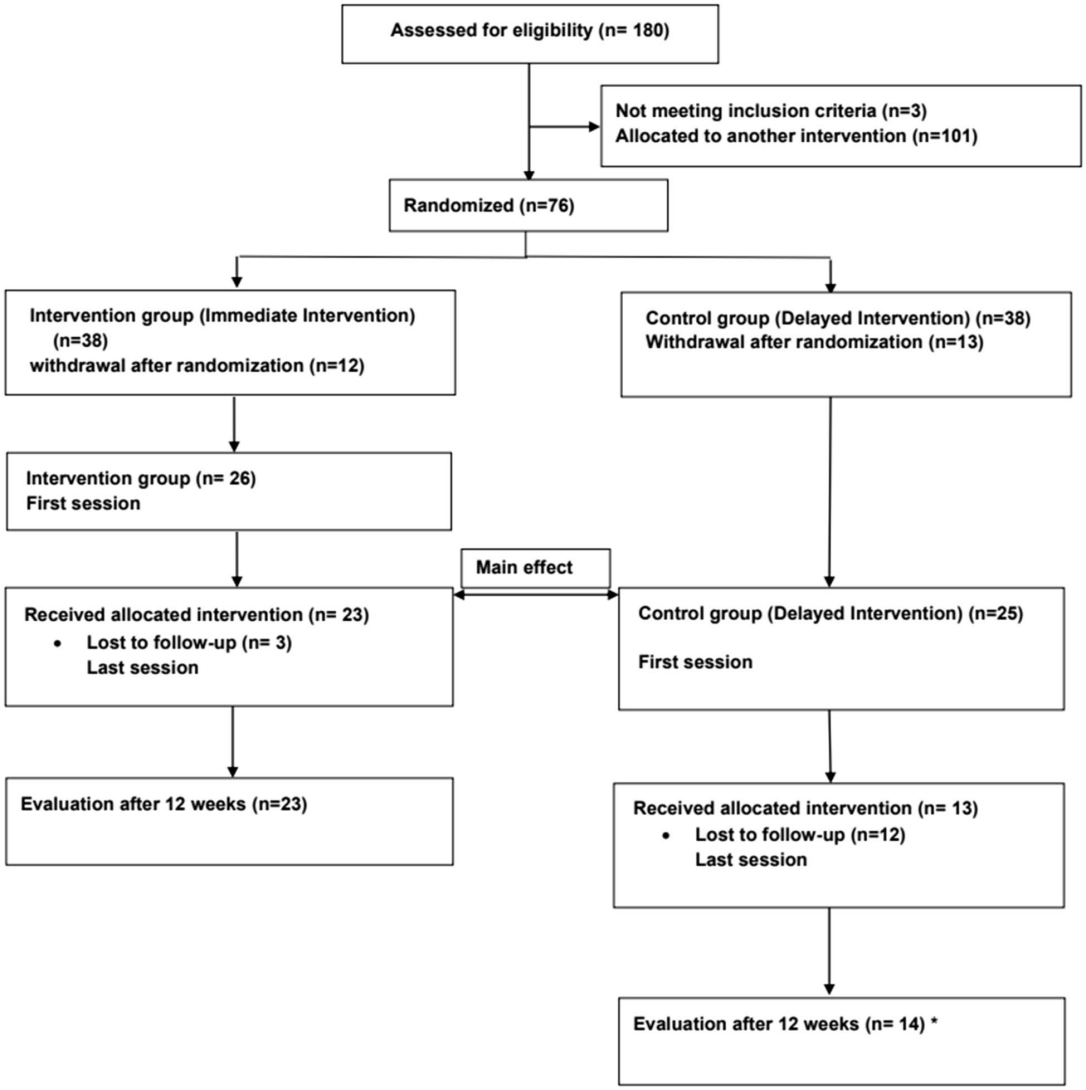
CONSORT flowchart

Syrian adult refugees (age ≥ 16 years) were recruited between 2018 and 2019, explained in detail in the protocol [25]. After participants were given written and verbal information in Arabic, those interested in participating answered a self-administered baseline questionnaire (Q0), including written informed consent. Of the 180 adults recruited, 76 had a predominant burden of mental health symptoms compared to pain disorders, and these were included in the TRT trial presented in this paper. Of these, 38 participants were randomized to the intervention group and 38 to the control group. As shown in the flow chart (Fig. 1), 26 participants (68%) from the intervention group and 28 participants (74%) from the control group attended the first session and completed the questionnaire on the day the group sessions began (Q1a). Twenty-three participants (61%) from the intervention group and 13 participants (34%) from the control group completed the same questionnaire (Q1b) six weeks later, at the end of the group sessions. Twenty-three participants (61%) from the intervention group and 14 participants (37%) from the control group completed the questionnaire (Q1c) 12 weeks after the intervention began. The number of participants who attended TRT sessions in the intervention and control groups by gender is summarized in (Additional file 1: Table A1).

### Measures

Two similar questionnaires were developed in Arabic, explained in detail in the protocol [25]. Q0 was used to identify baseline participants and included socio-demographic and migration-related information and health status, including mental health and chronic pain. Q1 was shorter and applied three times: at the start of the intervention (Q1a), at the end of the intervention six weeks later (Q1b), and again 12 weeks after the first intervention session (Q1c).

Impact of Event Scale-Revised (IES-R) is a self-report questionnaire of 22 items with a five-point scale (0–4) evaluating subjective distress caused by traumatic events in adult populations. There are three sub-scales: intrusion (intrusive thoughts, nightmares, intrusive feelings and imagery, dissociative-like re-experiencing), avoidance (numbing of responsiveness, avoidance of feelings, situations, and ideas), and hyperarousal (anger, irritability, hypervigilance, difficulty concentrating, and heightened startle). The total score used as the primary outcome for the RCT is calculated as the sum of all the 22 items (range 0–88). The General Health Questionnaire (GHQ-12) scale is designed for the general population, asking if the respondent has recently encountered a specific symptom or behaviour recently. GHQ-12 is among the most common and widely used screening instruments to measure mental health. It consists of 12 items, each using a 4-point scale (from 0 to 3) (less than usual, no more than usual, rather more than usual, or much more than usual) to determine the extent of a mental disorder over the past few weeks. The total score is calculated as the sum of the items with a range from 0 to 36, the higher scores indicating worse conditions. The BPI and GHQ-12 questionnaires were already validated in Arabic [27–29], and IES-R was validated in English. We translated the IES-R from English to Standard Modern Arabic by two double professional translators [30, 31]. After our study, the IES-R has been validated by another research group [32].

The Brief Pain Inventory- short form (PBI) which assesses pain over the last six months, including four items on pain intensity (questions about worst pain, least pain, average pain, pain right now), and seven items on how pain interferes with daily life (such as general activity, mood, walking ability, normal work, relations with other people, sleep, and enjoyment of life). Participants can score from 0 to 10 for each of the BPI items [33]. The score used as the secondary outcome in the current study was calculated as the average of the four items on pain severity.

### Inclusion and exclusion criteria

Participants who reported experiencing traumatic events and scored over 24 on the Impact of Event Scale–Revised (IES-R) were included in the TRT intervention. We also measured general mental health using the General Health Questionnaire-12 (GHQ-12). According to the protocol, the psychologists in the team assessed those participants who had high scores on either IES-R (37 or higher) or GHQ-12 (25 or higher) to determine the suitability of group therapy since such scores could indicate serious mental health problems. All the participants with high scores as explained were found suitable for TRT based on clinical evaluation conducted by the psychologists. The exclusion criteria included distance from place of residence to therapy locations and mandatory medical follow-ups (e.g. health complications from diabetes or cancer treatment), but no participant was excluded for these reasons.

### Outcomes

The primary outcome is mental health measured by IES-R. The secondary outcomes are general mental health measured by GHQ-12 and pain disorders measured by Brief Pain Inventory-Short form (BPI). The intervention effect was calculated according to the intention-to-treat (ITT) principle, comparing scores right after the intervention group had finished the intervention and immediately before the control group received the delayed intervention. In addition, we conducted a longitudinal analysis of change during the intervention process, for all participants and separated by gender, including the changes in the control group during the delayed intervention, for the same outcomes.

### Randomization and blinding

Block randomization was performed using a 1:1 allocation ratio with block sizes 4, 6 and 8 generated by a statistician using a rollac command in Stata version 15. The intervention was not blinded for either participants, instructors, or authors. The first author recruited the participants and analysed outcomes but had no access to the list of randomizations.

### Intervention

The Centre for Crisis Psychology at the University of Bergen organized a seminar to train all the health professionals and collaborating interpreters involved in the TRT intervention. Interpreters that were part of TRT intervention learned the specific terminology used during TRT intervention. The original intervention with children included five sessions. The TRT manual was modified for adults in this study with relevant examples and homework and was organized in six sessions. The objective of the extra session was to establish good group dynamics, explain the content and intention of the course and the reactions to stress and trauma, including traumatic experiences, reactions, and reminders. A consultant group of male and female Syrians advised us on how to adapt the implementation of the TRT to this particular group, especially with regard to gender issues, the necessity of text message reminders prior to each session, and session timing [25]. Two members of the team, working in pairs and with prior experience working with refugees, led the TRT sessions. TRT sessions were provided weekly for six weeks; one session lasts approximately two and a half hours with up to 10 participants. The first session addressed intrusive thoughts and feelings, the second session was about arousal, and the last sessions dealt with avoidance. The sessions were conducted for men and women separately, in Norwegian with an Arabic interpreter of the same gender as the group, who were well prepared beforehand. The systematic observation of the intervention sessions by the first author included taking detailed notes to evaluate whether the intervention followed the initial plan and to track behaviour in the groups, as explained in the protocol [25]. This was done at least twice in each group during the intervention period.

### Statistical analysis

Baseline characteristics for the included individuals are presented as mean and standard deviations for continuous variables and counts and percentages for categorical variables.

The ITT principle was used to calculate the intervention effect for all outcomes, which means that all the 76 individuals who originally gave their consent to participate and were randomized were included, even if they declined thereafter to attend when the intervention started. For all outcomes, the effect of the intervention was assessed using linear mixed models with random intercept for individuals and the outcome variable as the dependent variable. Data were analysed in long format (two observations per individual) with the score at Q0 as the first measurement for everyone. The second measurement was defined as the measurement at the last treatment session (six weeks) in the intervention group and the measurement at the first session for the control group. The intervention effect was estimated as the interaction effect between a binary group variable with the control group as the reference and a binary time variable with Q0 as the reference. The model also contained the main effect for the binary time variable as a covariate, while the main effect for group allocation was omitted. By omitting the main effect of group from the model, we achieve an adjustment for baseline differences in IES-R [34]. The intervention effect was reported as regression coefficients for the interaction term with 95% confidence intervals. It can be interpreted as the mean difference in change in outcome score after six weeks between the intervention and the control group after adjustment for potential differences in outcome at baseline. All individuals had baseline measurements (Q0), but 25 participants were lost to follow-up before the first session and had missing values on the second measurement. The linear mixed model approach in long format provides unbiased estimates of the intervention effect if follow-up data are missing at random, given the covariates included in the model. To investigate the presence of any bias because of differential loss to follow-up, we conducted sensitivity analysis with adjustment for variables that were significantly different between participants with complete data and participants who were lost to follow-up. As a second sensitivity analysis we also repeated the models with random intercept and slope for group membership, in addition to random intercept for individuals to investigate if differences in group dynamics within each recruiting wave could influence the results.

In addition to the estimation of intervention effects, we also conducted longitudinal analyses of changes during the intervention for both intervention and control (delayed intervention) groups combined. We used data in long format with three observations per person (first session, last session at six weeks and 12 weeks after first session) and applied linear mixed effects regression with random intercept for each individual. The inclusion of random slope for time did not improve the model fit and we, therefore, only estimated fixed effect for time. Time was modelled both as a categorical covariate with the first session as the reference and as a continuous covariate with the values 0, 6 and 12 to test for linear trend over weeks. Differences in change in outcomes over time between genders were investigated by stratification and inclusion of an interaction term between time and gender.

Stata SE version 16 was used to analyse the data. All tests have been two-sided, with 5% as the level of significance.

## Results

The baseline characteristics of the participants assigned to the intervention and control groups (n = 76) are summarized in Table 1. Participants in both groups were similar: young, predominantly Arab males, and approximately half of them were married and had children. We did not find any differences between the intervention and control groups at baseline in either IES-R, GHQ-12 or BPI scores. Also, the groups were balanced in terms of exposure to stressful events, daily use of medication and self-reported health.

**Table 1:** Characteristics of the intervention & control groups at baseline

		Intervention group	Control group
Total		38	38
Age (years), Mean (SD)		33 (10.4)	33 (10.7)
Female, N (%)		12 (32)	16 (42)
Ethnicity, N (%)	Arab	25 (66)	26 (68)
	Kurd	12 (32)	13 (34)
Stayed in any transit country, N (%)		26 (68)	23 (61)
Marital status (married), N (%)		19 (50)	24 (63)
Have children, N (%)		22 (57)	19 (50)
Number of children, Mean (SD)		1.6 (1.9)	1.9 (2.0)
Education (years), Mean (SD)		10 (4.8)	10 (4.4)
Self-reported health, N (%)	Poor	11 (29)	9 (24)
	Neither	15 (39)	14 (37)
	Good	12 (32)	15 (39)
Self-reported diseases and daily use of medication, N (%)			
Physical or psychological illness that impairs daily life at least 1 year		15 (39)	18 (47)
Physical pain more > 6 months		13 (34)	19 (50)
Never do exercise		20 (53)	20 (53)
Rheumatic arthritis		5 (13)	3 (8)
Joint disease		9 (24)	13 (34)
Mental health problems you have sought help for		16 (42)	9 (24)
Headache		14 (37)	13 (34)
Daily use of painkillers		5 (13)	4 (11)
Daily use of psychotropics		3 (8)	1 (3)
Study outcomes			
Impact Event Scale Revised (IES-R), Mean (SD)	Intrusion (8–32)	17 (5.9)	16 (6.3)
	Avoidance (8–32)	19 (5.3)	19 (4.9)
	Hyper-arousal (6–24)	13 (4.6)	13(4.9)
Exposure to stressful events, N (%)		38 (100)	38 (100)
IES-R scores ≥ 37, N (%)		30 (79)	28 (74)
BPI scores	Having pain today (yes), N (%)	29 (76)	32 (84)
	Pain intensity (1–10), Mean (SD)	3.6 (1.9)	3.6 (1.7)
	Pain interference (1–10), Mean (SD)	4.4 (1.9)	3.8 (2.3)
GHQ-12 (0–36), Mean (SD)		17 (6.5)	15 (6.9)
GHQ-12 scores ≥ 25, N (%)		5 (13)	3 (8)

From the 76 randomized participants, 51 had follow-up data on main and secondary outcomes (26 in the intervention and 25 in the control groups). A comparison of baseline characteristics between the 51 participants with two measurements and the 25 drop-out participants with only baseline measurement is reported in (Additional file 1: Table A2). Those who dropped out were younger than the actual participants, had stayed less often in a transit country before migrating to Norway, and had lower pain levels.

Table 2 shows mental health scores (IES-R and GHQ-12) and chronic pain (BPI) at baseline and after treatment for the intervention group, and at baseline and at the end of the waiting period for the control group, and the effect of the intervention. IES-R scores were significantly reduced by 7.5 and 6.0 points in the intervention and control groups, respectively. However, the ITT intervention effect comparing results after treatment for the intervention group and right before the delayed intervention for the control group, after adjustment for baseline IES-R measurements, was not significant with a regression coefficient B (95%CI) of − 1.3 (− 8.7, 6.2). The scores of GHQ-12 decreased significantly only in the intervention group during the 6-week period. The ITT analyses comparing the intervention group against the control group after adjustment for baseline GHQ-12 measurement showed the effect of the intervention on general mental health, with a B (95% CI) of -3.8 (− 7.2, − 0.4). BPI scores for pain intensity were unchanged in both groups.

**Table 2:** Effect of TRT intervention on the primary and secondary outcomes. Intention to treat analyses using linear mixed models

	Intervention, n = 38			Control, n = 38			Intervention effect	
	Baseline (Q0) Mean (SD)	Last session (6 weeks) (Q1b) Mean (SD)	p-value*	Baseline (Q0) Mean (SD)	End of waiting period (6 weeks) (Q1a) Mean (SD)	p-value*	B (95% CI) **	p-value
IES-R	47.8 (13.6)	40.3 (12.8)	0.002	47.0 (13.8)	41.0 (17.0)	0.014	− 1.3 (− 8.7, 6.2)	0.74
GHQ-12	17.1 (6.5)	10.7 (5.2)	< 0.001	15.0 (7.0)	14.0 (7.0)	0.253	− 3.8 (− 7.2, − 0.4)	0.02
BPI	3.6 (1.9)	3.6 (2.2)	0.594	3.6 (1.7)	3.6 (2.0)	0.687	− 0.01 (− 0.99, 0.97)	0.98

*Paired t-test for within-group change

**Regression coefficient for interaction term between group allocation and time

**Fig. 2:**
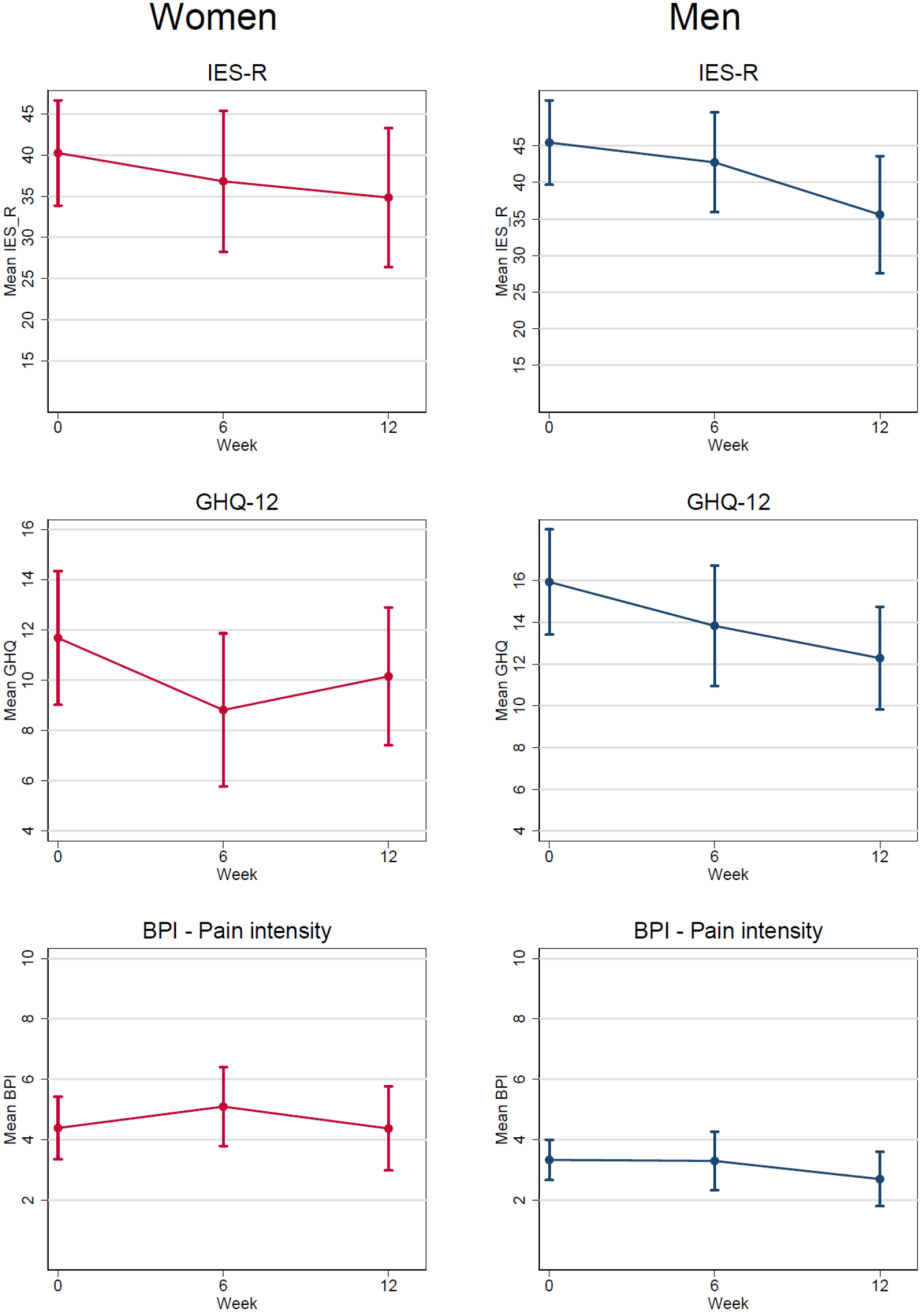
The longitudinal change in mean levels of IES-R, GHQ-12 and BPI

Figure 2 shows the longitudinal change in mean levels of IES-R, GHQ-12 and BPI for all participants (separately for men and women) (n = 76), including intervention and post-intervention periods for the control group (delayed intervention after having waited for treatment) and six weeks thereafter. For IES-R and GHQ-12 there was a significant reduction from the first session (week 0) until the last session (week 6) for men and women together, and the measurements in week 12 were also significantly reduced compared to week 0 (Additional file 1: Table A3). The same trends, although with broader confidence intervals, were applicable for each gender separately. The test for linear change with week treated as a continuous covariate was significant for men and women together and for men alone for both main outcomes. There was a significant reduction in pain scores for men; this was not the case for women alone or when women and men were combined.

## Discussion

TRT statistically improved general mental health among Syrian refugees, measured by GHQ-12, but did not improve trauma-oriented mental health symptoms assessed by IES-R. However, both intervention and control groups showed a positive trend in longitudinal analysis during the intervention phase in both mental health outcomes. TRT did not help reduce the chronic pain.

TRT was originally developed for children, and there are no previous studies of the effect of this intervention among adults for us to compare our results with. A review of the literature of different group interventions (narrative exposure therapy, cognitive behavioural therapy or CBT) in treating mental health problems among adult refugees showed generally positive effects of such interventions but pointed out the lack of randomization and the absence of control groups in the studies [35–37]. TRT in a previous Swedish study decreased symptoms of PTSD among unaccompanied refugee minors with PTSD. The Children’s Revised Impact of Event Scale (CRIES-8) and the Montgomery-Åsberg Depression Rating Scale–Self Assessment (MADRS-S) were the instruments for assessment [37]. Also, a cluster randomized trial using TRT decreased symptoms of depression measured through the Birleson Depression Self-Rating Scale (DSRS) among traumatized young migrants in Australia [38].

The differences in the results depending on the questionnaires used may be key to understanding why we find an effect in one of the questionnaires but not the other. The IES-R items measure mental health related to prolonged post-traumatic events [39], while the GHQ-12 items assess a person’s current status in terms of symptoms within the spectrum of common emotional disorders (i.e. depression and anxiety) and problems with everyday functioning [40, 41]. The effect of TRT might imply that this intervention better targets mental health problems relating to everyday-life situations, as opposed to symptoms clearly related to previous traumatic events. Another possible explanation for not finding an effect on the IES-R scale is fluctuating trauma symptoms. We may have chosen trial participants at a time when their symptoms were not sufficiently high at baseline. It is also possible, though less probable, that the IES-R scores would decrease in both groups because of regression to the mean, since this does not happen with symptoms measured by the GHQ-12. We observed improvement in both groups over time in the longitudinal analyses during the intervention time period and the first weeks after the end of the intervention. However, since we do not compare against a control group in these analyses, we cannot know the definite cause for this reduction in IES-R scores. The reduction in IES-R scores in the control group before the intervention (Table 2) indicates that this change could be a result of time or other events taking place besides our intervention. Another reason for why we did not find effect of TRT among adults, could be that the participants were not exposed to triggers of traumatizing events during the TRT sessions (in contrast to more typical exposure therapy). Exposure therapy has been shown to be effective for posttraumatic symptoms [42].

Our hypothesis on a secondary effect on pain levels was not confirmed. This could be because of a real lack of effect or because the intervention period was too brief. Another possible explanation is that a high pain level was not a requirement for inclusion in the study, and therefore we do not see a drop in BPI [43].

Attendance in some intervention sessions was low, especially for the female group. Earlier literature points to busy schedules, with everyday activities including compulsory school and working activities for refugees, but also the stigmatizing beliefs towards the treatment of mental illness [19, 44]. Being conscious of the possibly that participants may perceive stigma associated with certain language, we attempted to address this in our intervention, by trying to avoid most of the terms and words directly associated with mental illness. For example, instead of saying depression, we used words like worried, sleeplessness, or a description or a related symptom.

## Strengths and limitations

Our study has several strengths. First, and to the best of our knowledge, this is the first trial studying the effect of a self-help group intervention using TRT among adult refugees with mental health symptoms. Second, we have included the necessary number of participants in a group that is considered difficult to reach. Third, the Arabic background of the resource persons involved in the project, coupled with meetings with interpreters to discuss vocabulary beforehand, and the use of outcome measures validated for Arabic-speaking refugees, reduced language, and cultural barriers. Fourth, close monitoring of sessions allowed us to understand the differences in dynamics between groups, especially with regard to gender, which could be further examined through statistical analyses. Lastly, there was strong and well-established collaboration with the various Norwegian organizations and municipalities, which facilitated recruitment and improves the chances for further implementation. The complementary use of both qualitative and quantitative methods to understand the effect of the intervention ensured and evaluated the fidelity of the intervention to the initial plans. To ensure fidelity to the intervention, each group was observed by the first author (who speaks Arabic) at least three times (2.5 h for TRT sessions) with the aim of capturing changes and processes after consent from the members of the group.

This study also has some limitations. It does not include clinically assessed outcomes and relies on self-reported data. TRT for adults was still not validated at the time of our study, although it has been used with adolescents in Sweden with good effect, and we had the approval of the Children and War Foundation. A new TRT manual for adults is being developed, but only after our study was underway. Thus, any further evaluation for adults should await a possible standardization of TRT version for adults. Low attendance at the TRT sessions probably limited the effect in ITT analysis. Also, the sample size of the study (n = 38 in each group) did not allow to investigate results stratified on number of attended sessions. However, a stratification has been done, but we did not find that the effect was stronger among those who participated in more sessions. We almost managed to reach the necessary sample size needed from the sample size calculation, this is to say 38 out of 39 participants [23], but it can be argued that the assumed effect of -13.1 points on the IES-R in the sample size calculation was too optimistic. With a larger sample size, we would also have been able to detect medium sized effects of e.g. 6–7 points. The observed effect estimate in our study was small and would be unlikely to be clinically relevant even if a larger sample size could show statistical differences.

## Conclusion

This study reports promising results for TRT as a feasible self-help group intervention to improve general mental well-being among adult refugees. For more trauma-oriented symptoms or pain, there were no clear intervention effects. Our research adds to the evidence base required to prepare focused and successful health-care programmes for a vulnerable group. There is a need to adapt such an intervention to the everyday life of the participants.

## Supplementary Information

**Additional file 1: Table A1.** Number of participants who attended TRT sessions in each intervention and control groups by gender. **Table A2.** Group comparisons on characteristics of follow-up and dropped out. **Table A3.** Change in outcomes from first to last session and six weeks after last session for intervention and control groups combined (n=76) using linear mixed models.

## Data Availability

Not applicable.

## Abbreviations

RCT: Randomized controlled trial
TRT: Teaching Recovery Techniques
IES-R: Impact of EventScale–Revised
GHQ-12: General Health Questionnaire-12
BPI: Brief Pain Inventory- short form
PTSD: Post-traumatic stress disorder
CBT: Cognitive behavioural therapy
Q0: Baseline questionnaire
Q1: Follow-up questionnaire
ITT: Intention-to-treat analysis
