# Supplementary material for: Effect of a self-help group intervention using Teaching Recovery Techniques to improve mental health among Syrian refugees in Norway: a randomized controlled trial
Source: Int J Ment Health Syst. 2022 Sep 6;16:47. doi: 10.1186/s13033-022-00557-4 (PMC9450394; doi:10.1186/s13033-022-00557-4)
Supplement: Supplementary file 1 — Additional file 1: Table A1. Number of participants who attended TRT sessions in each intervention and control groups by gender. Table A2. Group comparisons on characteristics of follow-up and dropped out. Table A3. Change in outcomes from first to last session and six weeks after last session for intervention and control groups combined (n=76) using linear mixed models. [file 13033_2022_557_MOESM1_ESM.docx]

**Additional file 1**

**Table A1.** Number of participants who attended TRT sessions in each intervention and control groups by gender

**
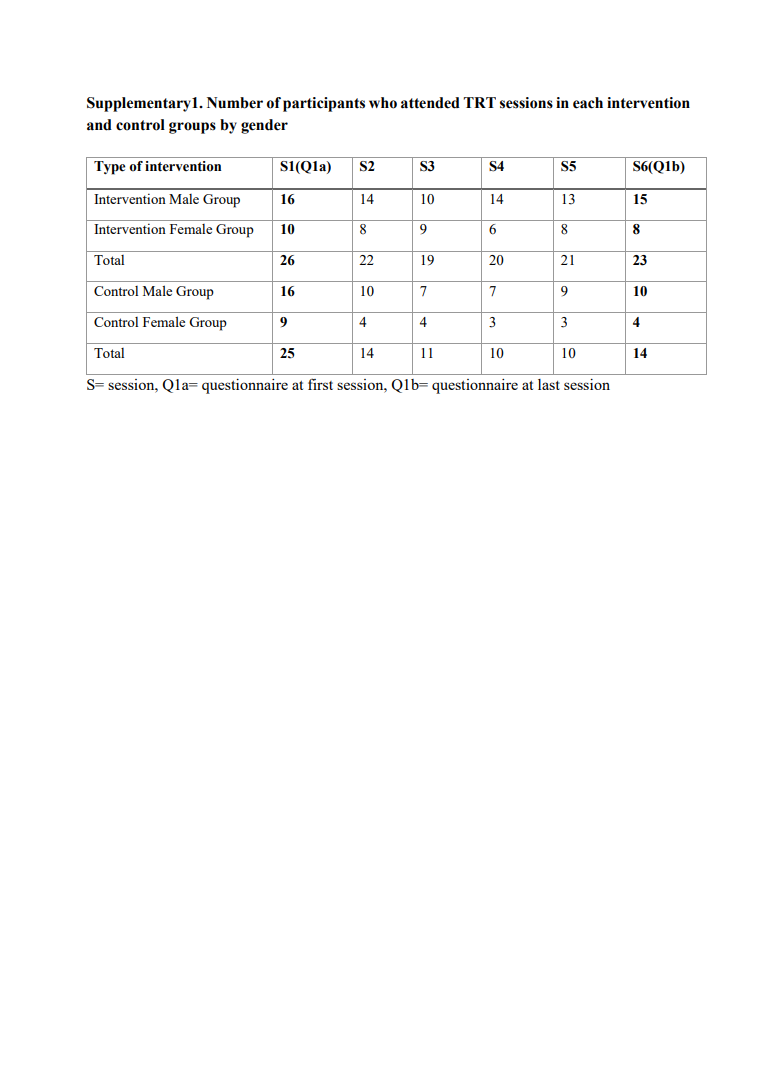
**

**Table A2.** Group comparisons on characteristics of follow-up and dropped out

|  | | | Follow-up | Dropped out | P-value |
| --- | --- | --- | --- | --- | --- |
| Total |  | | 51 | 25 |  |
| Age (years), Mean (SD) | | | 34.0 (10.3) | 29.0 (10.1) | 0.05 |
| Female, N (%) | | | 17 (33) | 11 (44) | 0.36 |
| Ethnicity, N (%) | Arab | | 36 (71) | 15 (60) | 0.35 |
|  | Kurd | | 15 (29) | 10 (40) |  |
| Stayed in a transit country on way to Norway, N (%) | | | 38 (75) | 11 (44) | 0.009 |
| Marital status (married), N (%) | | | 32 (63) | 11 (44) | 0.12 |
| Have children, N (%) | | | 30 (59) | 11 (44) | 0.22 |
| Number of children, Mean (SD) | | | 2.0 (2.0) | 1.2 (1.7) | 0.09 |
| Education (years), Mean (SD) | | | 9.8 (4.6) | 10.6 (4.6) | 0.45 |
| Exposure to stressful events, N (%) | | | 51 (100) | 25 (100) | 1.0 |
| Self-reported health, N (%) | | Poor | 16 (31) | 4 (16) | 0.35 |
|  |  | Neither | 18 (35) | 11 (44) |  |
|  |  | Good | 17 (33) | 10 (40) |  |
| Self-reported diseases and daily use of medication, N (%) | | | | | |
| Physical or psychological pain at least 1year | | | 23 (45) | 10 (40) | 0.67 |
| Physical pain more >6 months | | | 27 (53) | 5 (20) | 0.006 |
| Never do exercise | | | 28 (55) | 12 (48) | 0.57 |
| Rheumatic arthritis | | | 5 (10) | 3 (12) | 0.76 |
| Joint disease | | | 16 (31) | 6 (24) | 0.50 |
| Mental health problems | | | 18 (35) | 7 (28) | 0.52 |
| Headache | | | 20 (39) | 7 (28) | 0.33 |
| Daily use of painkillers | | | 6 (12) | 3 (12) | 0.77 |
| Daily use of psychotropics | | | 1 (2) | 3 (12) | 0.09 |
| Study outcomes | | | | | |
| Impact Event Scale Revised IES-R (0-88), Mean (SD) | Intrusion (8-32) | | 17.0 (6.3) | 16.0 (5.6) | 0.46 |
|  | Avoidance (8-32) | | 19.0 (4.9) | 17.7 (5.5) | 0.31 |
|  | Hyper-arousal (6-24) | | 13.0 (4.9) | 11.0 (4.2) | 0.12 |
| BPI scores | Having pain today (yes), N (%) | | 43 (84) | 18 (72) | 0.20 |
|  | Pain intensity (1-10), Mean (SD) | | 3.8 (1.7) | 3.1 (1.9) | 0.13 |
|  | Pain interference (1-10), Mean (SD) | | 4.5 (2.2) | 3.3 (1.7) | 0.02 |
| GHQ-12 (0-36) | | | 16.7 (7.4) | 14.7 (5.1) | 0.22 |

**Table A3.** Change in outcomes from first to last session and six weeks after last session for intervention and control groups combined (n=76) using linear mixed models.

|  |  | **Week 0** | **Week 6** | **Week 12** | **P-trend** |
| --- | --- | --- | --- | --- | --- |
|  |  |  | B (95% CI) | B (95% CI) |  |
| **Total** |  |  |  |  |  |
| **IES-R** |  | 0 (ref) | -6.0 (-10.2, -1.9) | -10.7 (-14.8, -6.6) | <0.001 |
| **GHQ-12** |  | 0 (ref) | -2.9 (-4.8, -1.0) | -3.3 (-5.2, -1.5) | <0.001 |
| **BPI** |  | 0 (ref) | -0.1 (-0.7, 0.5) | -0.6 (-1.2, 0.0) | 0.059 |
| **Women** |  |  |  |  |  |
| **IES-R** |  | 0 (ref) | -6.0 (-12.2, 0.3) | -6.5 (-12.4, -0.6) | 0.032 |
| **GHQ-12** |  | 0 (ref) | -3.3 (-6.0, -0.5) | -1.8 (-4.4, 0.8) | 0.185 |
| **BPI** |  | 0 (ref) | 0.4 (-0.5, 1.2) | -0.1 (-0.9, 0.7) | 0.846 |
| **Men** |  |  |  |  |  |
| **IES-R** |  | 0 (ref) | -6.4 (-11.5, -1.2) | -13.0 (-18.3, -7.7) | <0.001 |
| **GHQ-12** |  | 0 (ref) | -2.8 (-5.2, -0.4) | -4.2 (-6.7, -1.7) | 0.001 |
| **BPI** |  | 0 (ref) | -0.2 (-1.0, 0.5) | -0.8 (-1.6, -0.0) | 0.043 |

P-valued for interaction tests by gender: IES-R: 0.27, GHQ-12: 0.35 and BPI: 0.41
